# 4-(4-Chloro­phen­yl)-8-methyl-2-oxo-1,2,5,6,7,8-hexa­hydro­quinoline-3-carbonitrile

**DOI:** 10.1107/S1600536811036142

**Published:** 2011-09-14

**Authors:** Abdullah M. Asiri, Abdulrahman O. Al-Youbi, Hassan M. Faidallah, Khadija O. Badahdah, Seik Weng Ng

**Affiliations:** b Chemistry Department, Faculty of Science, King Abdulaziz University, PO Box 80203 Jeddah, Saudi Arabia; a Center of Excellence for Advanced Materials Research, King Abdulaziz University, PO Box 80203 Jeddah, Saudi Arabia; c Department of Chemistry, University of Malaya, 50603 Kuala Lumpur, Malaysia

## Abstract

The six-membered *N*-heterocyclic ring of the title compound, C_17_H_15_ClN_2_O, is fused with a methyl-substituted cyclo­hexene ring. The approximately planar nitro­gen-bearing ring (r.m.s. deviation 0.019 Å) is aromatic, and the N atom shows a trigonal–planar coordination; its benzene substituent is aligned at 77.1 (1) °. The cyclo­hexene ring adopts a half-chair conformation. In the crystal, inversion-related mol­ecules are linked by pairs of N—H⋯O hydrogen bonds, generating dimers.

## Related literature

For a related compound, see: Asiri *et al.* (2011 ▶).
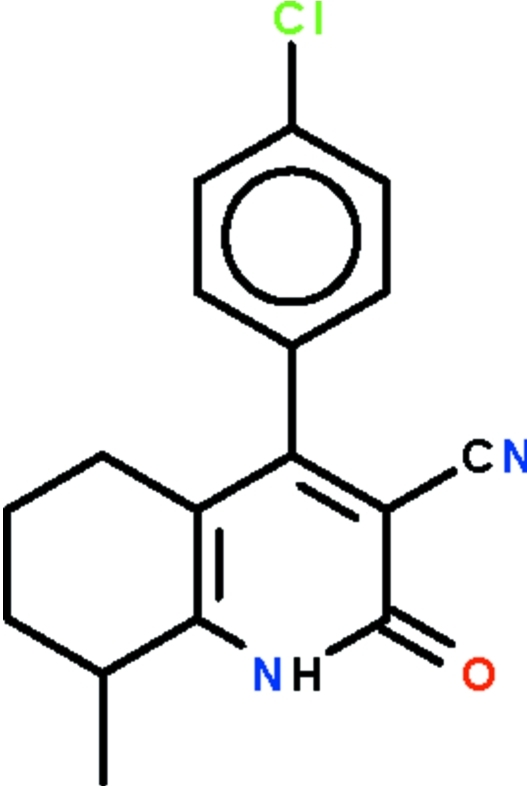


## Experimental

### 

#### Crystal data

C_17_H_15_ClN_2_O
*M* *_r_* = 298.76
Monoclinic, 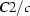

*a* = 18.6304 (4) Å
*b* = 18.7399 (4) Å
*c* = 8.5209 (2) Å
β = 90.229 (2)°
*V* = 2974.89 (11) Å^3^
*Z* = 8
Cu *K*α radiation
μ = 2.27 mm^−1^
*T* = 100 K
0.30 × 0.03 × 0.03 mm

#### Data collection

Agilent SuperNova Dual diffractometer with Atlas detector
Absorption correction: multi-scan (*CrysAlis PRO*; Agilent, 2010 ▶) *T*_min_ = 0.550, *T*_max_ = 0.935
10387 measured reflections
3014 independent reflections
2682 reflections with *I* > 2σ(*I*)
*R*_int_ = 0.025

#### Refinement

*R*[*F* ^2^ > 2σ(*F* ^2^)] = 0.065
*wR*(*F* ^2^) = 0.187
*S* = 1.03
3014 reflections
194 parameters
H atoms treated by a mixture of independent and constrained refinement
Δρ_max_ = 0.79 e Å^−3^
Δρ_min_ = −0.40 e Å^−3^

### 

Data collection: *CrysAlis PRO* (Agilent, 2010 ▶); cell refinement: *CrysAlis PRO*; data reduction: *CrysAlis PRO*; program(s) used to solve structure: *SHELXS97* (Sheldrick, 2008 ▶); program(s) used to refine structure: *SHELXL97* (Sheldrick, 2008 ▶); molecular graphics: *X-SEED* (Barbour, 2001 ▶); software used to prepare material for publication: *publCIF* (Westrip, 2010 ▶).

## Supplementary Material

Crystal structure: contains datablock(s) global, I. DOI: 10.1107/S1600536811036142/xu5320sup1.cif

Structure factors: contains datablock(s) I. DOI: 10.1107/S1600536811036142/xu5320Isup2.hkl

Supplementary material file. DOI: 10.1107/S1600536811036142/xu5320Isup3.cml

Additional supplementary materials:  crystallographic information; 3D view; checkCIF report

## Figures and Tables

**Table 1:** Hydrogen-bond geometry (Å, °)

*D*—H⋯*A*	*D*—H	H⋯*A*	*D*⋯*A*	*D*—H⋯*A*
N1—H1⋯O1^i^	0.91 (4)	1.84 (4)	2.744 (3)	174 (4)

Symmetry code: (i) 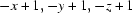.

**Fig. 1.**
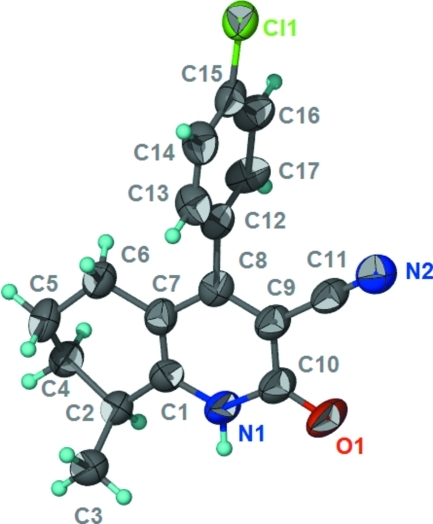
Thermal ellipsoid plot (Barbour, 2001) of C17H15ClN2O at the 70% probability level; hydrogen atoms are drawn as spheres of arbitrary radius.

## supplementary crystallographic information

### Comment

We have reported the synthesis of 2-oxo-4-phenyl-1,2,5,6-tetrahydrobenzo[*h*]quinoline-3-carbonitrile by using the reaction of benzaldehyde, 1-tetralone and ethyl cyanoacetate. The last reactant is incorporated into the product to form a part of the six-membered nitrogen-bearing ring, which is now endowed with an exocyclic cyanide group (Asiri *et al.*, 2011). In the present study, the use of 2-methylcyclohexane leads to the formation of the analogous compound with a cyclohexene ring fused with the six-membered nitrogen-bearing ring (Scheme I). The planar nitrogen-bearing ring (r.m.s. deviation 0.019 Å) is aromatic, and the N atom shows trigonal planar coordination; its benzene substituent is aligned at 77.1 (1) °. The cyclohexene ring adopts a half-chair conformation (Fig. 1). Two molecules are linked about a center-of-inversion by an N–H···O hydrogen bond to generate a dimer (Table 1).

### Experimental

4-Chlorobenzaldehyde (1.4 g, 10 mmol), 2-methylcyclohexanone (1.2 g, 10 mmol), ethyl cyanoacetate (1.1 g, 10 mmol) and ammonium acetate (6.2 g, 80 mmol) were heated in ethanol (50 ml) for 6 h. The solid product was collected, washed with water and then recrystallized from ethanol.

### Refinement

Carbon–bound H-atoms were placed in calculated positions [C–H 0.95–0.99 Å; *U*_iso_(H) 1.2–1.5 *U*_eq_(C)] and were included in the refinement in the riding model approximation. The amino H-atom was located in a difference Fourier map and was freely refined.

### Crystal data

**Table:**

C_17_H_15_ClN_2_O	*F*(000) = 1248
*M**_r_* = 298.76	*D*_x_ = 1.334 Mg m^−^^3^
Monoclinic, *C*2/*c*	Cu *K*α radiation, λ = 1.54184 Å
Hall symbol: -C 2yc	Cell parameters from 4602 reflections
*a* = 18.6304 (4) Å	θ = 3.3–74.1°
*b* = 18.7399 (4) Å	µ = 2.27 mm^−^^1^
*c* = 8.5209 (2) Å	*T* = 100 K
β = 90.229 (2)°	Prism, colorless
*V* = 2974.89 (11) Å^3^	0.30 × 0.03 × 0.03 mm
*Z* = 8	

### Data collection

**Table:**

Agilent SuperNova Dual diffractometer with Atlas detector	3014 independent reflections
Radiation source: SuperNova (Cu) X-ray Source	2682 reflections with *I* > 2σ(*I*)
Mirror	*R*_int_ = 0.025
Detector resolution: 10.4041 pixels mm^-1^	θ_max_ = 74.3°, θ_min_ = 3.4°
ω scan	*h* = −22→23
Absorption correction: multi-scan (*CrysAlis PRO*; Agilent, 2010)	*k* = −12→23
*T*_min_ = 0.550, *T*_max_ = 0.935	*l* = −10→10
10387 measured reflections	

### Refinement

**Table:**

Refinement on *F*^2^	Primary atom site location: structure-invariant direct methods
Least-squares matrix: full	Secondary atom site location: difference Fourier map
*R*[*F*^2^ > 2σ(*F*^2^)] = 0.065	Hydrogen site location: inferred from neighbouring sites
*wR*(*F*^2^) = 0.187	H atoms treated by a mixture of independent and constrained refinement
*S* = 1.03	*w* = 1/[σ^2^(*F*_o_^2^) + (0.0973*P*)^2^ + 6.1309*P*] where *P* = (*F*_o_^2^ + 2*F*_c_^2^)/3
3014 reflections	(Δ/σ)_max_ = 0.001
194 parameters	Δρ_max_ = 0.79 e Å^−^^3^
0 restraints	Δρ_min_ = −0.40 e Å^−^^3^

### Fractional atomic coordinates and isotropic or equivalent isotropic displacement parameters (Å^2^)

**Table:**

	*x*	*y*	*z*	*U*_iso_*/*U*_eq_	
Cl1	0.03066 (4)	0.40727 (4)	1.04897 (10)	0.0561 (3)	
O1	0.43887 (13)	0.55097 (12)	0.6145 (3)	0.0732 (8)	
N1	0.43862 (13)	0.43226 (13)	0.5593 (3)	0.0513 (6)	
N2	0.28356 (14)	0.58993 (14)	0.8353 (3)	0.0520 (6)	
C1	0.41066 (14)	0.36551 (15)	0.5654 (4)	0.0448 (6)	
C2	0.44737 (15)	0.31216 (15)	0.4544 (4)	0.0458 (6)	
H2	0.4536	0.3360	0.3503	0.055*	
C3	0.52222 (16)	0.29166 (17)	0.5165 (4)	0.0531 (7)	
H3A	0.5514	0.3348	0.5291	0.080*	
H3B	0.5455	0.2594	0.4418	0.080*	
H3C	0.5175	0.2677	0.6182	0.080*	
C4	0.39803 (15)	0.24814 (15)	0.4311 (3)	0.0461 (6)	
H4A	0.4254	0.2088	0.3818	0.055*	
H4B	0.3584	0.2615	0.3590	0.055*	
C5	0.36656 (18)	0.22215 (15)	0.5866 (3)	0.0498 (7)	
H5A	0.4062	0.2102	0.6598	0.060*	
H5B	0.3382	0.1783	0.5679	0.060*	
C6	0.31816 (16)	0.27916 (15)	0.6619 (4)	0.0480 (7)	
H6A	0.2711	0.2795	0.6074	0.058*	
H6B	0.3097	0.2664	0.7731	0.058*	
C7	0.35036 (15)	0.35264 (14)	0.6542 (3)	0.0425 (6)	
C8	0.31531 (15)	0.41183 (14)	0.7254 (3)	0.0402 (6)	
C9	0.34494 (15)	0.47924 (15)	0.7151 (3)	0.0453 (6)	
C10	0.41041 (17)	0.49152 (16)	0.6296 (4)	0.0532 (7)	
C11	0.31115 (15)	0.54044 (16)	0.7834 (3)	0.0451 (6)	
C12	0.24447 (15)	0.40529 (14)	0.8035 (3)	0.0396 (6)	
C13	0.23840 (17)	0.38018 (16)	0.9559 (3)	0.0480 (7)	
H13	0.2797	0.3628	1.0093	0.058*	
C14	0.17279 (18)	0.38018 (16)	1.0307 (3)	0.0512 (7)	
H14	0.1688	0.3630	1.1352	0.061*	
C15	0.11297 (16)	0.40541 (14)	0.9520 (3)	0.0436 (6)	
C16	0.11705 (16)	0.43002 (17)	0.8006 (4)	0.0504 (7)	
H16	0.0753	0.4465	0.7474	0.060*	
C17	0.18293 (16)	0.43036 (18)	0.7268 (3)	0.0502 (7)	
H17	0.1865	0.4479	0.6225	0.060*	
H1	0.481 (2)	0.439 (2)	0.508 (5)	0.078 (12)*	

### Atomic displacement parameters (Å^2^)

**Table:**

	*U*^11^	*U*^22^	*U*^33^	*U*^12^	*U*^13^	*U*^23^
Cl1	0.0605 (5)	0.0437 (4)	0.0642 (5)	−0.0013 (3)	0.0181 (4)	0.0010 (3)
O1	0.0666 (14)	0.0474 (13)	0.106 (2)	−0.0271 (11)	0.0384 (14)	−0.0337 (13)
N1	0.0412 (13)	0.0416 (13)	0.0711 (17)	−0.0104 (10)	0.0060 (12)	−0.0181 (12)
N2	0.0544 (14)	0.0540 (15)	0.0478 (13)	−0.0066 (11)	0.0023 (11)	−0.0180 (11)
C1	0.0362 (13)	0.0401 (14)	0.0580 (16)	−0.0042 (11)	−0.0058 (11)	−0.0078 (12)
C2	0.0416 (14)	0.0358 (13)	0.0599 (17)	−0.0031 (11)	0.0022 (12)	0.0042 (12)
C3	0.0464 (16)	0.0499 (17)	0.0631 (18)	−0.0040 (13)	0.0025 (13)	0.0048 (14)
C4	0.0467 (14)	0.0404 (14)	0.0510 (16)	−0.0046 (12)	0.0032 (12)	−0.0013 (12)
C5	0.0692 (19)	0.0329 (13)	0.0472 (15)	−0.0062 (12)	0.0010 (13)	0.0046 (11)
C6	0.0484 (15)	0.0383 (14)	0.0573 (16)	−0.0057 (11)	−0.0037 (12)	0.0050 (12)
C7	0.0445 (14)	0.0358 (13)	0.0472 (14)	−0.0024 (11)	−0.0113 (11)	−0.0001 (11)
C8	0.0444 (14)	0.0418 (14)	0.0342 (12)	−0.0054 (11)	−0.0083 (10)	−0.0001 (10)
C9	0.0460 (14)	0.0415 (14)	0.0484 (15)	−0.0097 (11)	0.0036 (11)	−0.0107 (11)
C10	0.0494 (15)	0.0440 (16)	0.0662 (19)	−0.0134 (12)	0.0103 (14)	−0.0193 (14)
C11	0.0469 (14)	0.0465 (15)	0.0420 (13)	−0.0132 (12)	0.0047 (11)	−0.0088 (12)
C12	0.0454 (14)	0.0373 (13)	0.0361 (12)	−0.0066 (10)	−0.0032 (10)	−0.0020 (10)
C13	0.0568 (16)	0.0489 (16)	0.0383 (14)	0.0081 (13)	−0.0004 (12)	0.0069 (12)
C14	0.0665 (18)	0.0461 (16)	0.0411 (14)	0.0045 (14)	0.0054 (13)	0.0093 (12)
C15	0.0534 (16)	0.0318 (13)	0.0457 (14)	−0.0067 (11)	0.0066 (12)	−0.0018 (10)
C16	0.0467 (15)	0.0563 (17)	0.0480 (15)	−0.0107 (13)	−0.0080 (12)	0.0046 (13)
C17	0.0463 (15)	0.0670 (19)	0.0374 (13)	−0.0133 (14)	−0.0068 (11)	0.0082 (13)

### Geometric parameters (Å, °)

**Table:**

Cl1—C15	1.745 (3)	C5—H5B	0.9900
O1—C10	1.241 (4)	C6—C7	1.503 (4)
N1—C1	1.356 (4)	C6—H6A	0.9900
N1—C10	1.368 (4)	C6—H6B	0.9900
N1—H1	0.91 (4)	C7—C8	1.424 (4)
N2—C11	1.150 (4)	C8—C9	1.382 (4)
C1—C7	1.378 (4)	C8—C12	1.486 (4)
C1—C2	1.539 (4)	C9—C11	1.433 (4)
C2—C4	1.524 (4)	C9—C10	1.442 (4)
C2—C3	1.538 (4)	C12—C13	1.386 (4)
C2—H2	1.0000	C12—C17	1.399 (4)
C3—H3A	0.9800	C13—C14	1.381 (4)
C3—H3B	0.9800	C13—H13	0.9500
C3—H3C	0.9800	C14—C15	1.382 (4)
C4—C5	1.531 (4)	C14—H14	0.9500
C4—H4A	0.9900	C15—C16	1.373 (4)
C4—H4B	0.9900	C16—C17	1.381 (4)
C5—C6	1.540 (4)	C16—H16	0.9500
C5—H5A	0.9900	C17—H17	0.9500
			
C1—N1—C10	125.7 (3)	C5—C6—H6B	109.1
C1—N1—H1	118 (3)	H6A—C6—H6B	107.8
C10—N1—H1	116 (3)	C1—C7—C8	118.3 (2)
N1—C1—C7	119.8 (3)	C1—C7—C6	120.7 (3)
N1—C1—C2	113.8 (2)	C8—C7—C6	120.7 (3)
C7—C1—C2	126.1 (2)	C9—C8—C7	120.1 (3)
C4—C2—C1	108.8 (2)	C9—C8—C12	117.4 (2)
C4—C2—C3	113.2 (2)	C7—C8—C12	122.4 (2)
C1—C2—C3	110.8 (2)	C8—C9—C11	122.0 (3)
C4—C2—H2	108.0	C8—C9—C10	121.1 (3)
C1—C2—H2	108.0	C11—C9—C10	116.8 (2)
C3—C2—H2	108.0	O1—C10—N1	121.2 (3)
C2—C3—H3A	109.5	O1—C10—C9	124.0 (3)
C2—C3—H3B	109.5	N1—C10—C9	114.8 (2)
H3A—C3—H3B	109.5	N2—C11—C9	178.6 (3)
C2—C3—H3C	109.5	C13—C12—C17	118.8 (3)
H3A—C3—H3C	109.5	C13—C12—C8	121.6 (2)
H3B—C3—H3C	109.5	C17—C12—C8	119.4 (2)
C2—C4—C5	111.7 (2)	C14—C13—C12	120.5 (3)
C2—C4—H4A	109.3	C14—C13—H13	119.7
C5—C4—H4A	109.3	C12—C13—H13	119.7
C2—C4—H4B	109.3	C13—C14—C15	119.3 (3)
C5—C4—H4B	109.3	C13—C14—H14	120.3
H4A—C4—H4B	107.9	C15—C14—H14	120.3
C4—C5—C6	111.5 (2)	C16—C15—C14	121.6 (3)
C4—C5—H5A	109.3	C16—C15—Cl1	119.4 (2)
C6—C5—H5A	109.3	C14—C15—Cl1	119.0 (2)
C4—C5—H5B	109.3	C15—C16—C17	118.8 (3)
C6—C5—H5B	109.3	C15—C16—H16	120.6
H5A—C5—H5B	108.0	C17—C16—H16	120.6
C7—C6—C5	112.5 (2)	C16—C17—C12	120.9 (3)
C7—C6—H6A	109.1	C16—C17—H17	119.5
C5—C6—H6A	109.1	C12—C17—H17	119.5
C7—C6—H6B	109.1		
			
C10—N1—C1—C7	−3.1 (5)	C7—C8—C9—C10	1.6 (4)
C10—N1—C1—C2	171.0 (3)	C12—C8—C9—C10	−174.6 (3)
N1—C1—C2—C4	−161.8 (3)	C1—N1—C10—O1	−178.3 (3)
C7—C1—C2—C4	11.8 (4)	C1—N1—C10—C9	−0.6 (5)
N1—C1—C2—C3	73.2 (3)	C8—C9—C10—O1	178.9 (3)
C7—C1—C2—C3	−113.2 (3)	C11—C9—C10—O1	1.0 (5)
C1—C2—C4—C5	−45.4 (3)	C8—C9—C10—N1	1.3 (4)
C3—C2—C4—C5	78.3 (3)	C11—C9—C10—N1	−176.6 (3)
C2—C4—C5—C6	63.7 (3)	C9—C8—C12—C13	−102.5 (3)
C4—C5—C6—C7	−43.5 (3)	C7—C8—C12—C13	81.5 (3)
N1—C1—C7—C8	5.8 (4)	C9—C8—C12—C17	72.3 (3)
C2—C1—C7—C8	−167.5 (3)	C7—C8—C12—C17	−103.8 (3)
N1—C1—C7—C6	179.6 (3)	C17—C12—C13—C14	−0.2 (4)
C2—C1—C7—C6	6.3 (4)	C8—C12—C13—C14	174.5 (3)
C5—C6—C7—C1	9.8 (4)	C12—C13—C14—C15	0.1 (5)
C5—C6—C7—C8	−176.5 (2)	C13—C14—C15—C16	0.5 (4)
C1—C7—C8—C9	−5.1 (4)	C13—C14—C15—Cl1	−178.4 (2)
C6—C7—C8—C9	−178.9 (3)	C14—C15—C16—C17	−1.0 (4)
C1—C7—C8—C12	170.9 (2)	Cl1—C15—C16—C17	178.0 (2)
C6—C7—C8—C12	−2.9 (4)	C15—C16—C17—C12	0.8 (5)
C7—C8—C9—C11	179.3 (3)	C13—C12—C17—C16	−0.2 (4)
C12—C8—C9—C11	3.1 (4)	C8—C12—C17—C16	−175.1 (3)

### Hydrogen-bond geometry (Å, °)

**Table:**

*D*—H···*A*	*D*—H	H···*A*	*D*···*A*	*D*—H···*A*
N1—H1···O1^i^	0.91 (4)	1.84 (4)	2.744 (3)	174 (4)

Symmetry codes: (i) −*x*+1, −*y*+1, −*z*+1.

## References

[bb1] Agilent (2010). *CrysAlis PRO* Agilent Technologies, Yarnton, England.

[bb2] Asiri, A. M., Faidallah, H. M., Al-Youbi, A. O., Alamry, K. A. & Ng, S. W. (2011). *Acta Cryst.* E**67**, o2468. 10.1107/S1600536811033873 PMC3200766 22065624

[bb3] Barbour, L. J. (2001). *J. Supramol. Chem.* **1**, 189–191.

[bb4] Sheldrick, G. M. (2008). *Acta Cryst.* A**64**, 112–122. 10.1107/S0108767307043930 18156677

[bb5] Westrip, S. P. (2010). *J. Appl. Cryst.* **43**, 920–925.

